# Supplementary material for: The causal role of circulating inflammatory markers in osteoporosis: a bidirectional Mendelian randomized study
Source: Front Immunol. 2024 Jul 18;15:1412298. doi: 10.3389/fimmu.2024.1412298 (PMC11291241; doi:10.3389/fimmu.2024.1412298)
Supplement: Supplementary file 1 [file DataSheet_1.zip › supplementary material/CIM-OSTEOPOROSIS /GCST90274802.csv_forest.pdf]

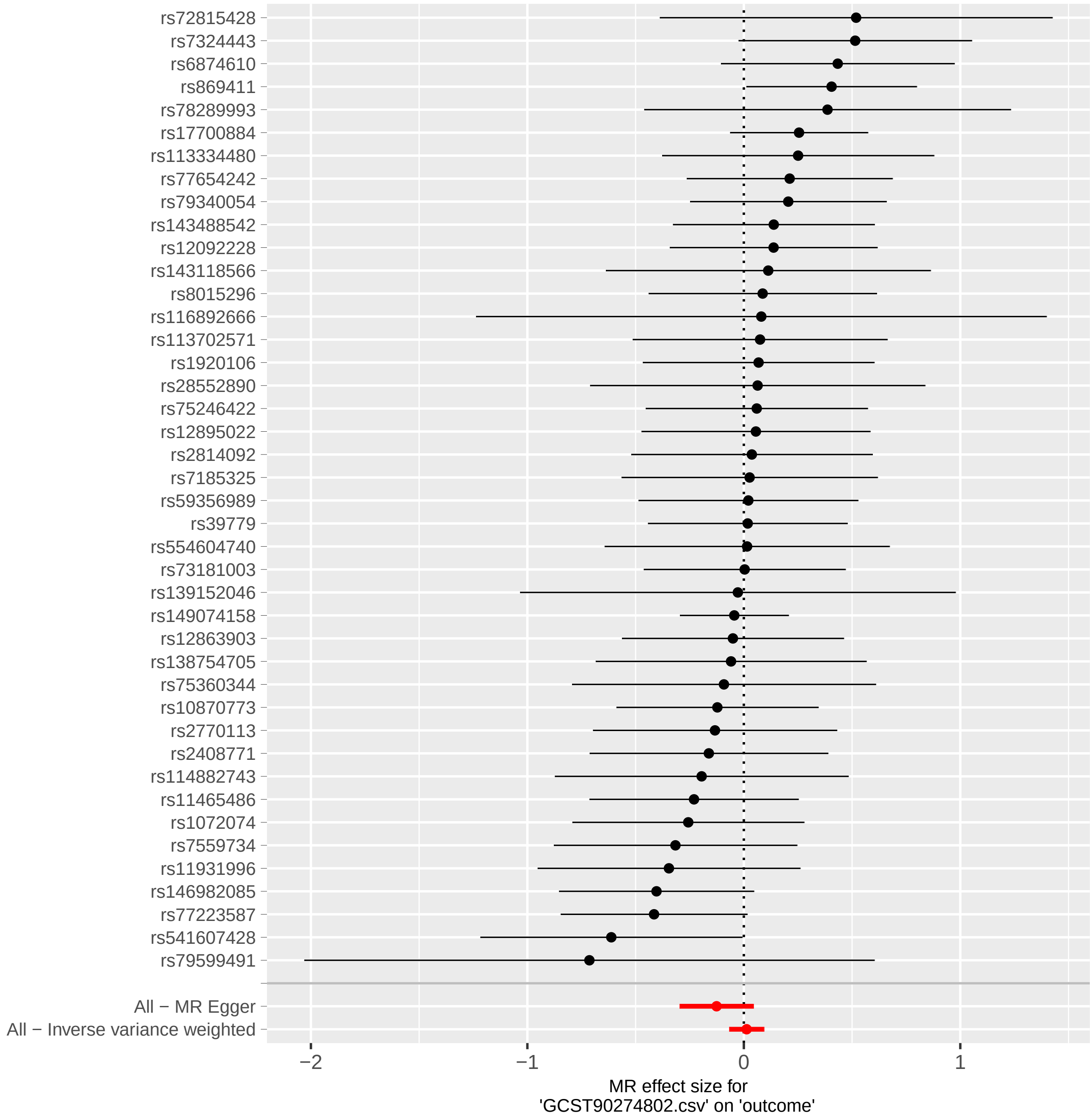

rs72815428
rs7324443
rs6874610
rs869411
rs78289993
rs17700884
rs113334480
rs77654242
rs79340054
rs143488542
rs12092228
rs143118566
rs8015296
rs116892666
rs113702571
rs1920106
rs28552890
rs75246422
rs12895022
rs2814092
rs7185325
rs59356989
rs39779
rs554604740
rs73181003
rs139152046
rs149074158
rs12863903
rs138754705
rs75360344
rs10870773
rs2770113
rs2408771
rs114882743
rs11465486
rs1072074
rs7559734
rs11931996
rs146982085
rs77223587
rs541607428
rs79599491
All − MR Egger
All − Inverse variance weighted
−2
−1
0
1
MR effect size for
'GCST90274802.csv' on 'outcome'
